# A PARP1-ERK2 synergism is required for the induction of LTP

**DOI:** 10.1038/srep24950

**Published:** 2016-04-28

**Authors:** L. Visochek, G. Grigoryan, A. Kalal, H. Milshtein-Parush, N. Gazit, I. Slutsky, A. Yeheskel, A. Shainberg, A. Castiel, R. Seger, M. F. Langelier, F. Dantzer, J. M. Pascal, M. Segal, M. Cohen-Armon

**Affiliations:** 1 Neufeld Cardiac Research institute, Sackler Faculty of Medicine, Tel-Aviv University, Tel-Aviv 69978, Israel; 2 Department of Neurobiology, the Weizmann Institute of Science, Rehovot 76100, Israel; 3 Department of Physiology and Pharmacology, Tel-Aviv University, Tel-Aviv 69978, Israel; 4 Sagol School of Neuroscience, Tel-Aviv University, Tel-Aviv 69978, Israel; 5 Bioinformatics Unit, George S. Wise Faculty of Life Sciences, Tel-Aviv University, Tel-Aviv 69978, Israel; 6 Mina and Everard Goodman Faculty of Life Sciences, Bar-Ilan University, 52900 Ramat Gan, Israel; 7 Cancer Research Center, Sheba Medical Center, 53621 Ramat Gan, Israel; 8 Department of Biological Regulation, the Weizmann Institute of Science, Rehovot 76100, Israel; 9 Department of Biochemistry and Molecular Biology, Sidney Kimmel Cancer Center, Thomas Jefferson University, Philadelphia, USA; 10 Biotechnology and Cell Signaling, UMR7242, Ecole Superieure de Biotechnologie Strasbourg, F-67400, Illkrich-Graffenstaden, France

## Abstract

Unexpectedly, a post-translational modification of DNA-binding proteins, initiating the cell response to single-strand DNA damage, was also required for long-term memory acquisition in a variety of learning paradigms. Our findings disclose a molecular mechanism based on PARP1-Erk synergism, which may underlie this phenomenon. A stimulation induced PARP1 binding to phosphorylated Erk2 in the chromatin of cerebral neurons caused Erk-induced PARP1 activation, rendering transcription factors and promoters of immediate early genes (IEG) accessible to PARP1-bound phosphorylated Erk2. Thus, Erk-induced PARP1 activation mediated IEG expression implicated in long-term memory. PARP1 inhibition, silencing, or genetic deletion abrogated stimulation-induced Erk-recruitment to IEG promoters, gene expression and LTP generation in hippocampal CA3-CA1-connections. Moreover, a predominant binding of PARP1 to single-strand DNA breaks, occluding its Erk binding sites, suppressed IEG expression and prevented the generation of LTP. These findings outline a PARP1-dependent mechanism required for LTP generation, which may be implicated in long-term memory acquisition and in its deterioration in senescence.

PolyADP-ribose polymerases (PARPs) catalyze an abundant post-translational modification of nuclear proteins by polyADP-ribosylation. In this modification, NAD (Nicotinamide adenine dinucleotide) derived ADP-ribosyl moieties form ADP-ribose polymers on glutamate, lysine and aspartate residues of PARPs and their substrates^1^,^2^. Binding of the most abundant nuclear polyADP-ribose polymerase PARP1 to DNA single-strand breaks activates the protein and thereby triggers DNA base-excision repair^1^,^2^.

Recent findings implicated PARP1 in additional processes in the chromatin, including gene expression regulated by chromatin remodeling, DNA methylation or recruitment of transcription factors^2^,^3^,^4^,^5^,^6^. Moreover, alternative mechanisms of PARP1 activation in the absence of DNA damage were identified in a variety of cell types and cell-free systems. They include PARP1 activation by a variety of signal transduction mechanisms inducing intracellular Ca^2+^ release and activation of phosphorylation cascades^2^,^7^,^8^,^9^.

Numerous findings implicated the phosphorylation of extracellular signal regulated kinase-2 (Erk2) in synaptic plasticity and long-term memory^10^,^11^,^12^. Interestingly, recent *in vivo* experiments also revealed a pivotal role of PARP1 activation in long-term memory acquisition during learning^13^,^14^,^15^,^16^,^17^,^18^, but the explicit molecular mechanism underlying this un-expected role of PARP1 has not been identified.

Here, we disclose a molecular mechanism in the chromatin of cerebral neurons, which is activated by stimulation-induced Erk-PARP1 binding and synergistic activity required for immediate early genes (IEG) expression implicated in long-term memory. Furthermore, identified intra-molecular re-arrangements in DNA-bound PARP1 preventing its binding to phosphorylated Erk2, interfered with stimulation-induced IEG expression and LTP generation in the presence of DNA single-strand breaks, usually accumulated in aged irreplaceable cerebral neurons^19^,^20^.

## Results

### PARP1-dependent long-term potentiation in the hippocampal CA3-CA1 connections

Long-term potentiation (LTP) in the hippocampal CA3-CA1 connections is currently used as a model for long-term memory^21^,^22^,^23^. In our experiments, field excitatory postsynaptic potentials (fEPSPs) were recorded from hippocampal slices of mice. Long-term potentiation in the hippocampal CA3-CA1 connections was induced by a brief high frequency stimulation of the Schaffer collaterals using two sets of bipolar electrodes placed on both sides and equidistant from the recording pipette, such that two independent stimulation channels were used for each slice (Methods).

**Figure 1:**
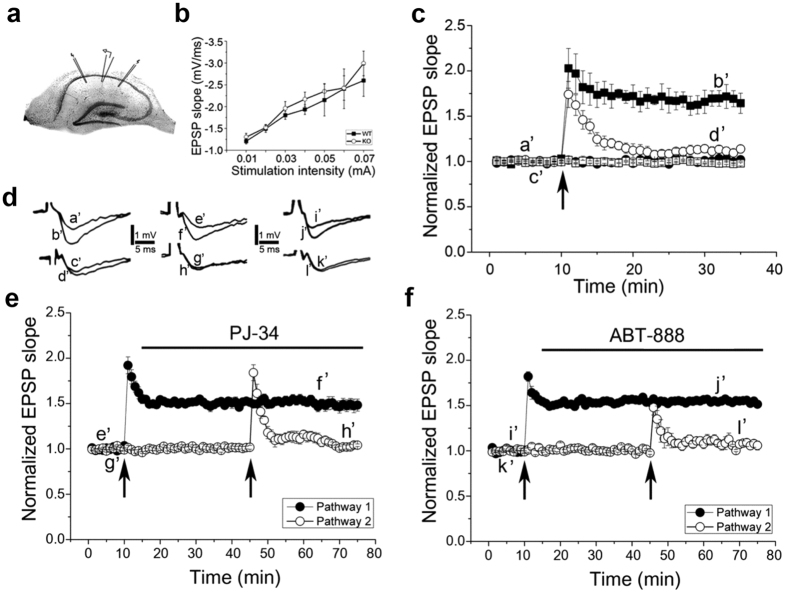
PARP1 is required for LTP generation in hippocampal slices. (**a**) A schematic diagram of the hippocampal slice with the two independent pathway stimulation and recording. (**b**) Input/output relations in response to stimulation of the Schaffer collateral system in CA1 region of the mouse hippocampal slice (Methods). No difference between slices of wild-type and PARP1 KO mice. (**c**) Normal LTP was measured in the hippocampus of WT mice (6 hippocampal slices prepared from 2 WT mice) in response to a high frequency (tetanic) stimulation (100 Hz, 1 sec) (⦁). In 6 hippocampal slices prepared from 2 PARP1 KO mice LTP was not generated by the same stimulation (⚬). (**d**) Sample illustration of individual records sampled at the indicated time intervals in (**c**,**e**,**f**). (**e**,**f**) PARP inhibitors prevented LTP generation in rat hippocampal slices (representative results obtained in 6 hippocampal slices prepared from 2 WT mice). Tetanic stimulation before application of PARP1 inhibitors PJ-34 and ABT-888 produced a sustained LTP. PJ-34 (**e**) and ABT-888 (**f**) did not affect the baseline activity, or the already potentiated responses, but completely prevented the generation of LTP in the pathway tested 30 min after their application. Arrowheads indicate applied stimulation.

To examine a possible effect of PARP1 on LTP, hippocampal slices were prepared from WT and PARP1 KO mice (Methods). LTP was generated in response to high frequency (100 Hz, 1 sec) tetanic stimulation in hippocampal slices of WT mice. However, there was a striking attenuation of the potential in the potentiated pathway in hippocampal slices of PARP1 KO mice. LTP was not generated in the hippocampal CA3-CA1 connections of PARP1-KO mice (Fig. 1a–c).

To examine a possible effect of PARP1 activity on LTP generation, PARP1 activity was blocked by the potent PARP inhibitors PJ-34 and ABT-888 (Fig. 1e,f, n = 7 and n = 5 slices, respectively). PJ-34 and ABT-888 were added at concentrations that inhibited polyADP-ribosylation of PARP1 in the cortex and hippocampus of rats^15^. PJ-34 and ABT-888 were added to the recording medium 5 min after tetanic stimulation to one pathway, and 30 minutes before similarly stimulating the second pathway (Methods; Fig. 1a,e,f). The tetanic stimulations produced a pathway-selective LTP before the application of PARP inhibitors (Methods). The first tetanic stimulation caused LTP, maintained for 70 minutes at 1.48 ± 0.004 and 1.54 ± 0.01 above baseline (average values calculated before application of PJ-34 and ABT-888, respectively). LTP induced in the first pathway was maintained stable even after application of PARP inhibitors. In contrast, tetanic stimulation delivered to the second pathway after 30 min perfusion of each PARP1 inhibitor failed to produce LTP (average values measured at the end of experiments with PJ-34 and ABT-888, 1.04 ± 0.01 and 1.07 ± 0.01 above baseline, respectively). Thus, each of the PARP inhibitors applied 30 min before stimulation completely prevented LTP development without affecting the already developed LTP or baseline responses. These results implicated PARP1 in the generation of LTP by tetanic stimulation of the Schaffer collaterals. The PARP1 inhibitors did not block nor attenuated excitatory postsynaptic NMDA current, which evokes LTP in the hippocampal CA3-CA1 connections^23^ (Fig. S1).

In view of a similar effect of MEK inhibitors on LTP generation in the hippocampal CA3-CA1 connections^24^ (Fig. S2), and accumulating findings implicating Erk-induced IEG expression in LTP and long-term memory aquisition^25^,^26^,^27^,^28^, we examined possible role of PARP1 in Erk-induced IEG expression.

### A PARP1-dependent immediate early gene expression in response to high frequency stimulation

Stimulation inducing LTP is restricted to a small subset of afferents in the hippocampus^21^,^23^. It was impossible to isolate the stimulated neurons for examining biochemical signals associated with LTP. To overcome this difficulty, we used a model system of cultured cerebral neurons stimulated by electrical stimulation (Methods). High frequency (tetanic) stimulation (3 repeats of a 100 Hz, 1 sec duration pulse, followed by a 10 sec pause) applied to cultured cerebral neurons caused synaptic potentiation, indicated by pre-synaptic vesicles recycling (Fig. S3), which induces post-synaptic excitatory currents and synaptic long-term potentiation^29^,^30^.

**Figure 2:**
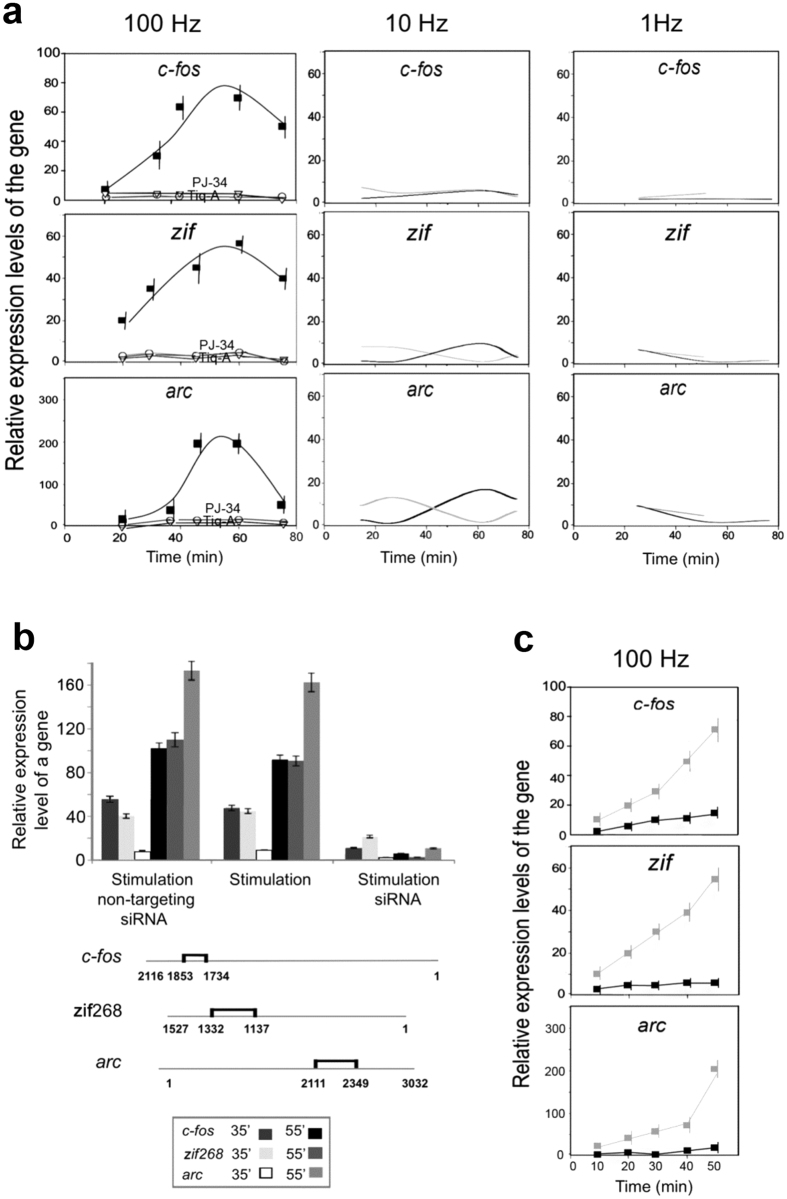
PARP1 mediated expression of immediate early genes *cfos, zif268* and *arc* in response to stimulation. (**a**) The relative expression rate of immediate early genes *c-fos, zif268* and *arc* was measured by RT-PCR at the indicated time intervals after stimulation of cultured rat cerebral neurons (3 repeats of 100 Hz, 10 Hz or 1 Hz stimulation, 1 sec duration, each followed by 10 sec pause). An enhanced expression rate of *c-fos, zif268* and *arc* was measured in response to the high frequency stimulation (100 Hz; black line), also causing pre-synaptic vesicle recycling, characterizing synaptic potentiation (Fig. S3). The stimulation-induced gene expression was suppressed in cerebral neurons treated with either of the PARP inhibitors PJ-34 (10 μM) and Tiq-A (50 μM) (grey lines). Each value represents the mean value with calculated variation coefficient (Standard deviation divided by the average value) of 4 separate reactions in each of 4 experiments. (**b**) The relative expression of *c-fos, zif268 and arc* measured by RT-PCR, 35 and 55 min after stimulation (100 Hz, 3 repeats, 1 sec each, 10 sec pause) was suppressed after PARP1 silencing by siRNA (72 hours, 150 nM). Each value represents the mean value (with calculated variation coefficient) of 4 separate reactions in each of 3 experiments. (**c**) The genes *c-fos, zif268* and *arc* were scarcely expressed within 50 min after stimulation (3 repeats 100 Hz, 1 sec, 10 sec pause) in cultured cerebral neurons of PARP1 KO mice (black lines and full squares). The relative expression rate of the genes in similarly stimulated cerebral neurons of WT mice is presented for comparison (grey line and full squares). Each value represents the average value of 4 separate reactions (with calculated variation coefficient) performed in each of 3 different experiments.

Stimulation-induced expression of the immediate early genes *c-fos, zif268* and *arc* that are implicated in LTP and long-term memory acquisition^25^,^26^,^27^,^28^, was measured by RT-PCR in stimulated cultured cerebral neurons, 8–10 days after plating (Fig. 2).

We found that only high frequency stimulation (3 repeats of a 100 Hz, 1 sec duration pulse, followed by a 10 sec pause) induced expression of *c-fos, zif268* and *arc* in the cultured cerebral neurons within minutes after stimulation (Fig. 2a). The expression of *arc* lagged after *zif268* expression, probably due to Zif268 (Egr1) acting as one of *arc* transcription factors^28^. Notably, the high frequency stimulation did not induce a non-specific Erk-dependent gene expression (eg., *cJun*^31^ was not expressed; Fig. S4).

The expression of *c-fos, zif268* and *arc* in response to the high frequency stimulation was suppressed in cerebral neurons treated with each of the PARP inhibitors PJ-34 (10 μM) and Tiq-A (50 μM). In addition, their expression was similarly suppressed after PARP1 silencing (by siRNA, 150 nM, 72 hours; Fig. 2a,b), or PARP1 genetic deletion in cerebral neurons of PARP1-KO mice (Fig. 2c). These results supported a possible implication of PARP1 in the stimulation-induced expression of *c-fos, zif268* and *arc* in the cerebral neurons. So, PARP inhibition, PARP1 silencing or its genetic deletion similarly interfered with stimulation-induced IEG expression in cerebral neurons and LTP induction in hippocampal CA3-CA1 connections (Figs 1 and 2).

A possible role of PARP1 activation in the recruitment of RNA-polII or transcription factors to the IEG promoters^32^ seemed unlikely in-view of recent evidence for RNA-polII poised in the promoter of *arc*^33^, and IEG transcription factors bound to CBP (CREB binding protein), with its HAT (histone acetyl-transferase) activity induced by their phosphorylation^34^. Instead, we examined a possible role of PARP1 in the phosphorylation of poised transcription factors, initiating the expression of *c-fos, zif268* and *arc* in response to stimulation.

### PARP1 binding to phosphorylated Erk2 and its activation in response to high frequency stimulation

**Figure 3:**
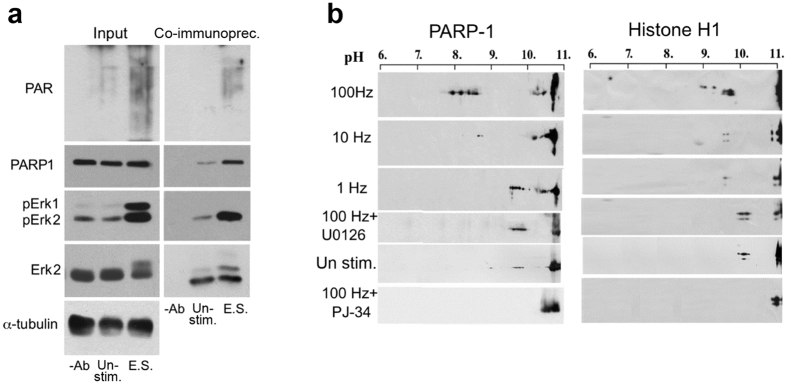
PARP1 binding to phosphorylated Erk2 and its activation in nuclei of cerebral neurons stimulated by high frequency stimulation. (**a**) Activated PARP1 co-immunoprecipitated with phosphorylated Erk2 by antibody directed against the c-terminal of Erk2 in nuclear protein extracts of electrically stimulated rat cerebral neurons (3 repeats of 1 sec pulse, 100 Hz frequency, each followed by 10 sec pause). PARP1 polyADP-ribosylation was detected by anti-PAR antibody (directed against polyADP-ribosyl moieties; Alexis). Phosphorylated Erk2 was immunolabeled by antibody directed against phosphorylated Erk1/Erk2 and by antibody directed against the c-terminal of Erk2 (Methods). Mounting control: α-tubulin. Representative results of 3 experiments are displayed. (**b**) PARP1 activation measured by its shifted isoelectric point (pI; pH shifted from 10.5 to 7.5) and by the shifted pI of its substrate histone H1 (from pH > 11 to 6.5) due to polyADP-ribosylation (Fig. S5; Methods) in nuclear extracts of stimulated cultured cerebral neurons (3 repeats of 1 sec pulse, 100 Hz, 10 sec pause). PARP1 and H1 were not similarly polyADP-ribosylated in unstimulated neurons, neurons stimulated by low frequency stimulation (1 Hz and 10 Hz), or neurons treated with either MEK or PARP inhibitors (10 μM U0126 or 10 μM PJ-34, respectively). Representative results of 4 experiments are displayed.

Transcription factors of *c-fos, zif268* and *arc* are activated by Erk-induced phosphorylation^34^,^35^,^36^,^37^,^38^. We therefore examined the effect of high frequency electrical stimulation on Erk phosphorylation. Rat brain cerebral neurons in primary cultures were stimulated by a variety of electrical stimulations (8–10 days after plating; Methods). Erk was phosphorylated in nuclei of cerebral neurons stimulated by a high frequency stimulation (3 repeats of a 100 Hz, 1 sec duration pulse, followed by a 10 sec pause), and phosphorylated Erk2 co-immunoprecipitated with PARP1 in nuclear protein extracts of the stimulated cerebral neurons (Fig. 3a). In addition, PARP1 and its prominent substrate linker histone H1, were highly polyADP-ribosylated (Fig. 3b). This finding was consistent with PARP1 activation in response to the high frequency electrical stimulation. PARP1 activation was identified by its immunolabeled ADP-ribose residues, and it was quantified by the shift in the isoelectric point (pI) of PARP1 and its substrate H1 towards lower pH concomitantly with its polyADP-ribosylation (Fig. 3b and S5; Methods). In un-stimulated neurons and in neurons stimulated by low frequency stimulations, Erk1/2 were hardly phosphorylated in the nuclear protein extracts, Erk2 did not co-immunoprecipitate with PARP1, and PARP1 and H1 were not polyADP-ribosylated (Fig. 3a,b). Furthermore, PARP1 activation by high-frequency stimulation was prevented in cerebral neurons treated with the specific MEK inhibitor U0126 (10 μM; Fig. 3b), similarly to PARP1 inhibition by its inhibitor PJ-34 (10 μM). These results support a linkage between PARP1 binding to phosphorylated Erk2 in the nuclear extracts and PARP1 activation in response to the high-frequency stimulation (further examined by bioinformatics methods), reminiscent of recombinant PARP1 activation by recombinant phosphorylated Erk2 in a cell-free system^9^.

Next, we examined possible effects of Erk-PARP1 binding on IEG expression in the stimulated cerebral neurons.

### Phosphorylated Erk2 bound to activated PARP1 was recruited to promoters of immediate early genes *c-fos* and *zif268* in response to high frequency stimulation

**Figure 4:**
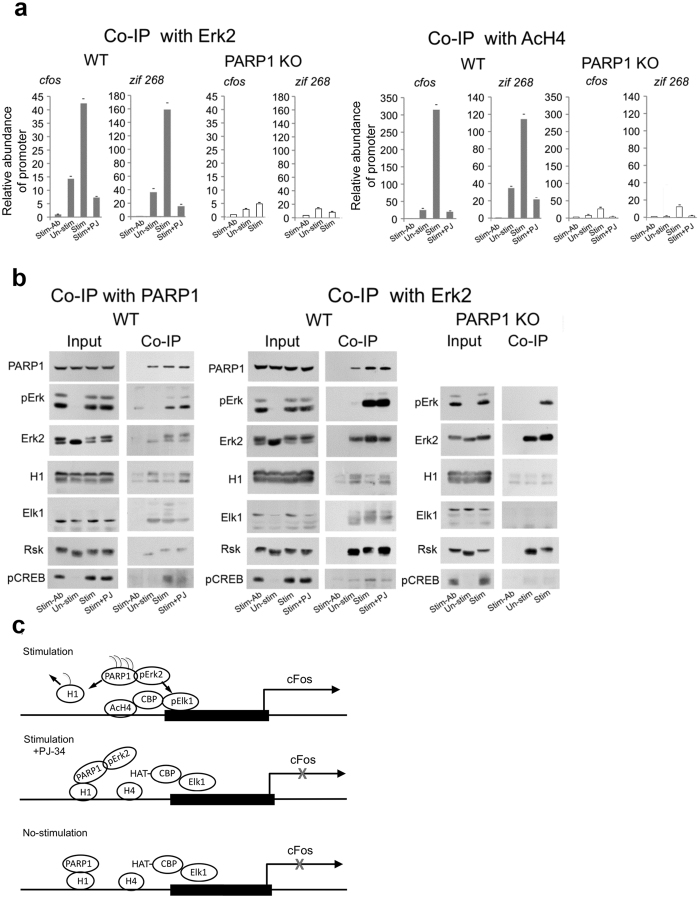
A PARP1-dependent recruitment of phosphorylated Erk2 to promoters of *c-fos* and *zif268.* (**a**) Recruited phosphorylated Erk2 and acetylated H4 (AcH4) to promoters of *c-fos* and *zif268* in the chromatin of stimulated cerebral neurons (chromatin was crosslinked after stimulation; ChIP assay). Cerebral neurons of WT and PARP1-KO mice were stimulated by 3 repeats of 1 sec 100 Hz stimulation followed by 10 sec pause. ***Left:*** DNA segments in the promoters of *c-fos* and *zif268* were amplified by RT-PCR after DNA isolation from crosslinked chromatin segments co-immunoprecipitated with phosphorylated Erk2 by antibody directed against the c-terminal of Erk2. ***Right:*** DNA segments in the promoters of *c-fos* and *zif268* were amplified by RT-PCR after DNA isolation from crosslinked chromatin segments co-immunoprecipitated with antibody directed against acetylated histone H4 (AcH4; Methods). Each value represents the mean abundance of co-immunoprecipitated promoter fragments measured by 4 different reactions (with calculated variation coefficient) in 4 different experiments. (**b**) Proteins recovered from the crosslinked chromatin segments of stimulated cerebral neurons (3 repeats of 1 sec 100 Hz stimulation, 10 sec pause) of WT and PARP1 KO mice co-immunoprecipitated with PARP1 or Erk2 antibodies. The displayed results indicate: PARP1 binding to phosphorylated Erk2 in the stimulated cerebral neurons of WT mice. PARP inhibition improved their binding to histone H1, but impaired their binding to Elk1and CREB. Phosphorylated Erk2 scarcely bound to Elk1 and CREB in the chromatin of stimulated PARP1-KO cerebral neurons. Representative results of 4 different experiments.(**c**) A schematic presentation of PARP1 dependent expression of immediate early gene, based on the results in panels (**a**,**b**) Binding of phosphorylated Erk2 to PARP1 induces its polyADP-ribosylation and release of its polyADP-ribosylated substrate, linker histone H1. This facilitates Erk-induced phosphorylation of transcription factor Elk1, hisone acetylation and gene expression.

We used the ChIP assay to identify recruited proteins to promoters of the immediate early genes *cfos* and *zif268* in response to stimulation. Chromatin cross-linking following stimulation (3 repeats of a 100 Hz, 1 sec pulse, followed by a 10 sec pause) revealed phosphorylated Erk2 and acetylated histone H4 co-immunoprecipitated with DNA segments in the promoters of *c-fos* and *zif268* in cerebral neurons of WT mice (Fig. 4a). In addition, PARP1 was bound to phosphorylated Erk2 in the chromatin segments, and PARP1 inhibition did not impair their binding (Fig. 4b). However, phosphorylated Erk2 and acetylated H4 hardly co-immunoprecipitated with the promoters of *cfos* and *zif268* after PARP1 inhibition, or PARP1 genetic deletion in stimulated cerebral neurons of PARP1-KO mice (Fig. 4a), indicating that binding of phosphorylated Erk2 to PARP1 was required for phosphorylated Erk2 access to the promoters of *cfos* and *zif268* in the stimulated cerebral neurons.

These results suggest that PARP1 binding to phosphorylated Erk2 inducing PARP1 activation^9^ (Fig. 3, and the effect of PARP1-Erk2 binding on PARP1 activation further examined by bioinformatics methods) and polyADP-ribosylation of the PARP1 substrate linker histone H1, may facilitate H1 release from the DNA^2^, rendering IEG promoters accessible to PARP1-bound phosphorylated Erk2 (Fig. 4b,c). In support, PARP1-bound to phosphorylated Erk2 did not co-immunoprecipitate with its substrate H1, unless polyADP-ribosylation was inhibited (Fig. 4b).

This outlines a possible synergism between Erk-induced PARP1 activation and polyADP-ribosylation of linker histone H1 facilitating recruitment of phosphorylated Erk2 to transcription factors of c*fos* and *zif268* (Fig. 4a). A PARP1-mediated phosphorylation of their transcription factors, inducing the HAT activity of CBP, and their binding to specific elements in the IEG promoters^34^,^35^ complies with co-immunoprecipitation of PARP-bound phosphorylated Erk2 and acetylated histone with DNA segments in the IEG promoters and with transcription factors Elk1 and CERB^34^,^35^,^36^,^37^,^38^ (Fig. 4a,b). Co-immunoprecipitation of phosphorylated Erk2 or acetylated H4 with DNA segments in the IEG promoters was prevented by PARP1 inhibition or its genetic deletion in cerebral neurons of PARP1-KO mice (Fig. 4a). In accordance, stimulation-induced expression of c*fos* and *zif268* was prevented by PARP1 inhibition or its genetic deletion (Fig. 2).

Notably, phosphorylated Erk co-immunoprecipitated with its cytoplasmic/nuclear substrate, Rsk (ribosomal S6 kinase)^36^ in the chromatin of both WT and PARP1 KO mice (Fig. 4b), suggesting a possible PARP1-independent Erk-induced gene expression via Rsk phosphorylation^36^ in PARP1 KO mice.

### Identified docking sites of phosphorylated Erk in PARP1

**Figure 5:**
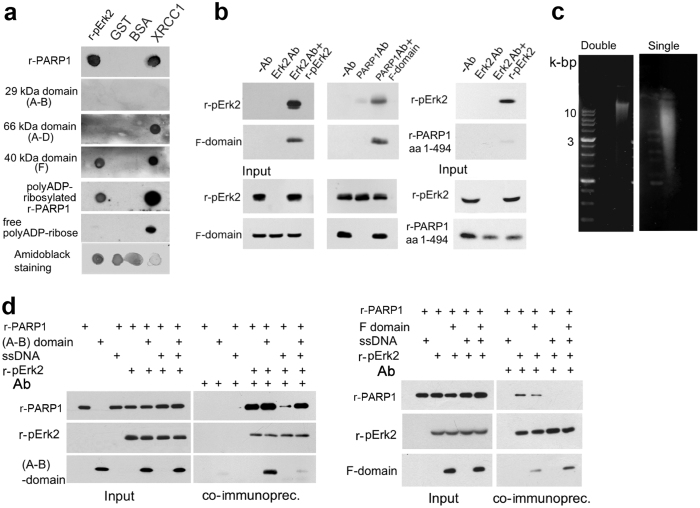
Identified Erk-binding domains in PARP1. (**a**) Erk-binding domains in recombinant PARP1 (r-PARP1) were identified by dot-blot analysis (Methods). Recombinant phosphorylated Erk2 (r-p-Erk2) bound to r-PARP1, to recombinant polyADP-ribosylated PARP1 and to the recombinant F-domain of PARP1 (aa656-1014) containing its catalytic site. r-p-Erk2 did not bind to the DNA-binding domain (A-B; aa1-201) of PARP1, nor to its (A–D) auto-modification domain (aa1-524) or ADP-ribose polymers. r-PARP1 did not bind to BSA (excluding non-specific binding of r-PARP1 to proteins), nor to GST (attached as a fusion protein to r-p-Erk2). The binding of XRCC1 to r-PARP1, polyADP-ribosylated r-PARP1 and ADP-ribose polymers served as a positive control. Representative results of 3 different experiments are displayed. (**b)** Testing the exclusive binding of the F-domain of PARP1 with r-p-Erk2. ***Left:*** Co-immunoprecipitation of r-p-Erk2 (200 ng) with the F-domain of PARP1 (200 ng) using antibody directed against the c-terminal of Erk2. Recombinant F-domain was detected with PARP1 antibody (Serotec, MCA1522). ***Middle:*** Co-immunoprecipitation of r-p-Erk2 (200 ng) with the F-domain (200 ng) immunoprecipitated by antibody directed against PARP1 (Alexis, ALX210-302). ***Right:*** The recombinant domain of PARP1 (aa1-494) was not co-immunoprecipitated with r-p-Erk2. Representative results of 3 different experiments are displayed. (**c**) Single strand DNA breaks detected in sheared DNA (ssDNA, Sigma; Methods). (**d**) Single-strand DNA breaks interfered with the binding of r-PARP1 to r-p-Erk2. ***Left:*** r-PARP1 (200 ng) did not co-immunoprecipitate with r-p-Erk2 (200 ng) in the presence of ssDNA (1 μg). Application of the recombinant DNA binding domain of PARP1 (A-B domain, 200 ng; immunolabeled by PARP1 antibody ALX210-302) restored PARP1-Erk2 binding. ***Right:*** The F-domain of PARP1 (200 ng) interfered with the co-immunoprecipitation of r-PARP1 with r-p-Erk2, even in the absence of ssDNA. Representative results of 4 different experiments are displayed.

**Figure 6:**
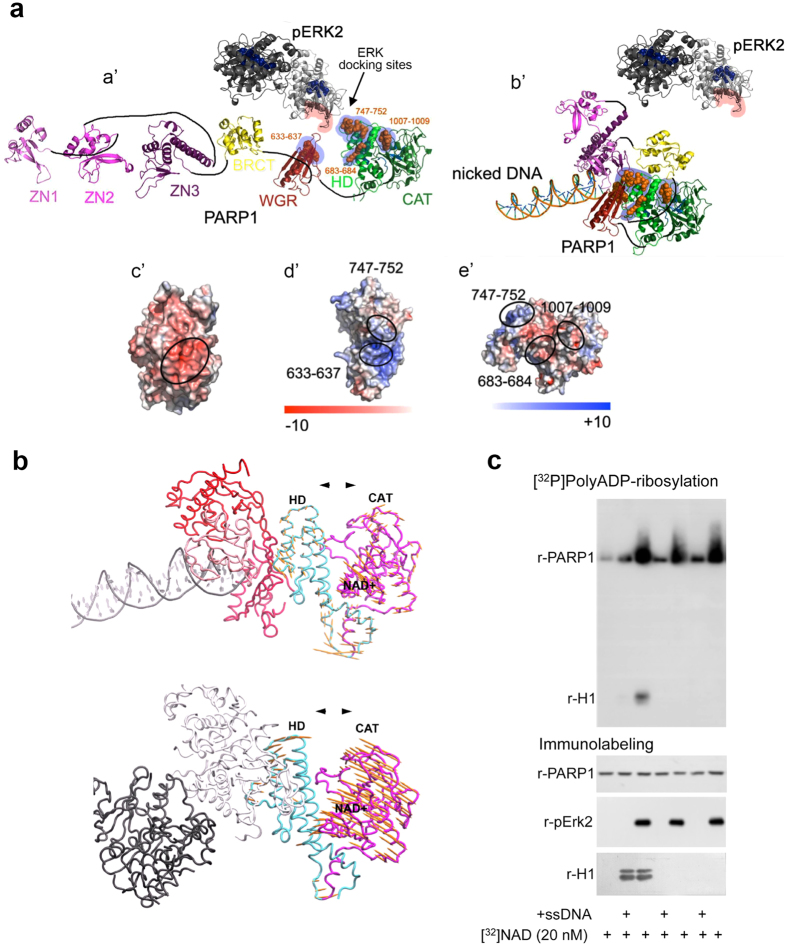
Intra-molecular re-arrangements in PARP1 associated with its activation. (**a**) Optional consensus Erk-docking sites in the F-domain of PARP1, which contains its catalytic (CAT), helical (HD) and WGR domains, are occluded in DNA-bound PARP1. (a’) A ribbon structural model for the open conformation of PARP1 with optional consensus docking sites for phosphorylated Erk. Phosphorylated Erk monomers (in homodimer) are indicated by dark and light gray ribbons. The optional Erk binding motifs (^633^KYPKK^637^, ^683^KK^684^, ^747^KKPPLL^752^ and ^1007^FNF^1009^) in HD, CAT and WGR domains of PARP1 are indicated by orange spheres. The CRS/CD protein-binding region in Erk2, and the optional Erk binding motifs in PARP1 are highlighted by red and blue shadows, to indicate negatively (red) and positively (blue) charged domains (c’–e’). (b’) The closed conformation of DNA-bound PARP1 was modeled according to protein data bank (PDB 4DQY). (c’) The electrostatic potential map of phosphorylated Erk2. The CRS/CD protein-binding region on phosphorylated Erk2 is indicated by a black circle. Negatively and positively charged domains are colored red and blue, respectively (see color bar in panels (**d**,**e**)). (d’,e’) The electrostatic potential map calculated for domains WGR, HD and CAT of PARP1 in the region containing consensus Erk docking motifs (circled). (**b)** Calculated intra-molecular motions in the helical (HD) and the catalytic domains (CAT) of PARP1 exposing its NAD binding site. Intra-molecular motions were calculated for region aa662-1014 in the complex of PARP1-bound to nicked DNA (Protein Data Bank 4DQY), as well as in PARP1-bound to phosphorylated Erk-homodimer. The localization of the binding site of NAD in the CAT-domain of PARP1 is indicated. A motion with the helical and the catalytic domains of PARP1 moving to opposite directions exposes the NAD binding site in Erk-bound PARP1 (Supplementary Methods; Movies displayed in Fig. S6). (**c**) A high [^32^P]polyADP-ribosylation of r-PARP1 incubated with r-phosphorylated Erk2 at low [^32^P]NAD concentration. At low [^32^P]NAD concentration(50 nM, 1 μCi/ sample), r-PARP1 (100 ng) incubated with r-p-Erk (100 ng) was more [^32^P]polyADP-ribosylated than r-PARP1 incubated with ssDNA (1μM). Recombinant H1 was [^32^P]polyADP-ribosylated by activated PARP1 in both reactions. The [^32^P]PolyADP-ribosylated proteins were auto-radiographed. Recombinant proteins were immunolabeled. Representative results of 4 different experiments.

We searched PARP1 domains for binding sites of Erk. Dot-blot analysis and co-immunoprecipitation of recombinant domains of PARP1 with recombinant phosphorylated Erk2 disclosed an exclusive binding of recombinant phosphorylated Erk2 to the F-domain of PARP1 (aa556-1014), which contains its WGR, helical (HD), and catalytic (CAT) ADP-ribosyl transferase domains^39^ (Figs 5a,b and 6a). Recombinant phosphorylated Erk2 did not bind to PARP1 domains containing its DNA binding sites (Zn1-Zn2), nor to the auto-modification domain of PARP1 (aa1-494) (Fig. 5a,b). Phosphorylated Erk2 did not bind to [^32^P]ADP-ribose polymers, and polyADP-ribosylation did not prevent the binding of recombinant PARP1 to recombinant phosphorylated Erk2 (Fig. 5a).

A non-specific binding of Erk2 to recombinant PARP1 and its recombinant domains was excluded, as well as a possible binding of PARP1 and its F-domain to GST (glutathione *S*-transferase) attached as a fusion protein to recombinant phosphorylated Erk2 (Fig. 5a).

As expected, XRCC1 (X-ray repair cross complementing protein-1) was bound to recombinant PARP1, polyADP-ribosylated PARP1 and ADP-ribose polymers^1^ (Fig. 5a).

Notably, recombinant PARP1 did not co-immunoprecipitate with recombinant phosphorylated Erk2 in the presence of DNA damaged by single strand breaks (ssDNA; Methods) (Fig. 5c,d). However, their binding was restored after applying the recombinant DNA-binding domain of PARP1 (recombinant A-B domain containing its zinc fingers Zn1, Zn2; aa1-201) (Fig. 5d), possibly due to PARP1 displacement from its binding sites in nicked DNA^40^ (Fig. 5d). Furthermore, the recombinant F-domain of PARP1 interfered with the binding of recombinant PARP1 to recombinant phosphorylated Erk2 even in the absence of ssDNA, possibly due to their competition for common binding sites in phosphorylated Erk2 (Fig. 5a,b,d).

Next, residues in the F-domain of PARP1 (aa556-1014) were searched for known MAP kinases docking motifs^41^,^42^,^43^. Four sites on PARP1 partially match the known docking sites of MAP kinases in various proteins: ^633^KYPKK^637^, ^683^KK^684^, ^747^KKPPLL^752^ and ^1007^FNF^1009^ (Fig. 6a). In the helical domain of PARP1, 683 KK 684 partially matches docking motif for Erk^41^, 747 KKPPLL 752 matches docking motif DEJL for Erk^42^. In the catalytic domain of PARP1, 1007 FNF 1009 partially matches DEF docking motif (FXFP) for Erk^43^, and 633 KYPKK 637 in the WGR domain of PARP1 matches docking motif DEJL for Erk^42^. The domains in positively charged patches in the F-domain of PARP1 (aa633-637 and aa747-752; Fig. 6a) may bind to Erk via its negatively charged protein binding domain (CRS/CD region)^41^,^44^ (Fig. 6a). Indeed, mutations in the CRS/CD region of recombinant phosphorylated Erk2 interfered with the activation of recombinant PARP1 by recombinant phosphorylated Erk2 in a cell-free system^9^.

Notably, the recently disclosed PARP1 structural re-arrangements accompanying its binding to DNA^39^ occlude the indicated consensus docking sites of phosphorylated Erk in the F-domain of DNA-bound PARP1 (Fig. 6a). This may explain the failure of DNA-bound PARP1 to bind phosphorylated Erk2 (Fig. 5d), while its binding to histone H1 and other PARP1 substrates is not affected^39^,^45^ (Fig. 6c).

### Intra-molecular dynamics in PARP1 bound to phosphorylated Erk2 can induce its activation

Intra-molecular dynamics in PARP1-bound to phosphorylated Erk2 homodimer was compared to intra-molecular dynamics in DNA-bound PARP1 by using the anisotropic network model (ANM)^46^ (http://ignmtest.ccbb.pitt.edu/cgi-bin/anm/anm1.cgi). This analysis was based on the potential Erk docking sites in the helical, catalytic and WGR domains of PARP1 (Fig. 6a), and on its predicted binding to homodimers of phosphorylated Erk in the nucleus^9^,^47^.

The resulting computed intra-molecular directions of motion in the combined complex of PARP1 bound to phosphorylated Erk2 expose the NAD binding site in the catalytic domain of PARP1 (Fig. 6b and S6). Exposure of its NAD binding site complies with the identified activation of Erk-bound PARP1 in stimulated cerebral neurons and in cell-free systems^9^ (Figs 3 and 6c, respectively).

The computed intra-molecular dynamics of PARP1 bound to phosphorylated Erk, exposing its NAD binding site anticipate polyADP-ribosylation of Erk-bound PARP1 (Fig. S6). This prediction is in consistence with the higher Erk-induced [^32^P]polyADP-ribosylation of recombinant PARP1 as compared to its DNA-induced [^32^P]polyADP-ribosylation at low [^32^P]NAD concentrations^9^ (Fig. 6c).

Thus, high frequency stimulation of cerebral neurons, inducing Erk phosphorylation and translocation to the nucleus^47^ may also induce PARP1 activation and PARP1-mediated IEG expression (Figs 2, 3, 4), unless the DNA is damaged by single strand breaks (Fig. 6). This notion was examined in stimulated cerebral neurons.

### PARP1 binding to single-strand DNA breaks interfered with IEG expression

**Figure 7:**
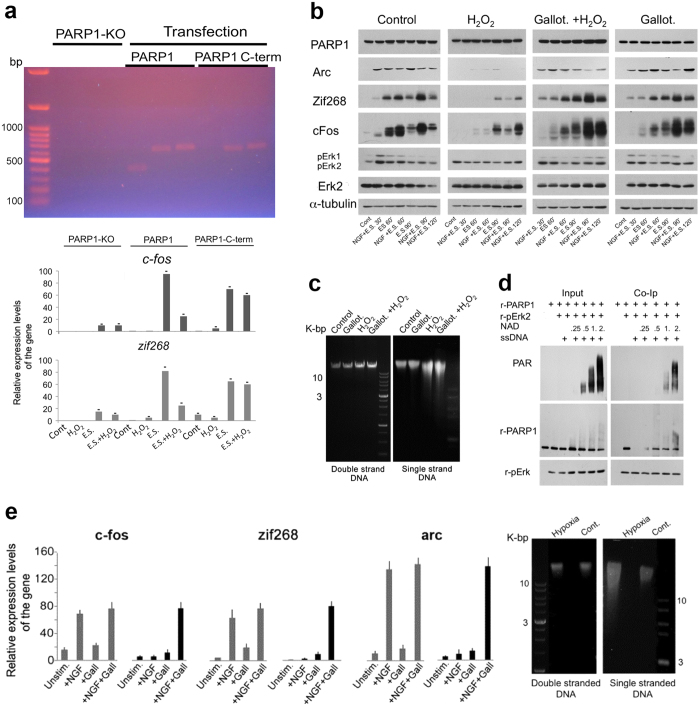
Stimulation-induced IEG expression in cerebral neurons was attenuated by PARP1 binding to nicked DNA. (**a**) Expression of *c-fos* and *zif268* in PARP1-KO cerebral neurons transfected with PARP1 constructs. ***Upper***: GFP-fusion vectors with constructs of either full-length PARP1 or PARP1 lacking its N-terminal (aa1-201) were expressed in PARP1-KO cerebral neurons (Methods). PARP1 expression was identified by three DNA segments, two encoding regions in the C-terminal (2132–2712, 2117–2717 bp) and one encoding region in the N-terminal of PARP1 (136–536 bp). ***Lower:*** Stimulation-induced (3 repeats of 100 Hz, 1 sec pulse, followed by 10 sec pause) expression of *c-fos* and *zif268* in the transfected neurons was measured by RT-PCR, without or after treatment with H_2_O_2_ (1 mM, 10 min) causing single strand breaks (**c**). This treatment attenuated the gene expression only in PARP1-KO neurons transfected with full-length PARP1. Each value represents the mean relative expression rate measured in 4 reactions performed in each of 3 experiments. (**b**) High levels of cFos, Zif268 and Arc proteins were measured in nuclear protein extracts of cultured cerebral neurons during 120 min after stimulation (3 repeats of 100 Hz, 1 sec, each followed by 10 sec pause), without or after incubation with NGF (60 ng/ml, 5 min). Protein levels were low in neurons treated with H_2_O_2_ (1 mM, 10 min) before stimulation, unless the neurons were pre-treated with the PARG inhibitor, gallotannin (100 μM, 60 min). Representative results of 3 experiments are displayed. (**c**) Gallotannin (100 μM, 60 min) did not induce DNA single-strand breaks repair in cerebral neurons treated with H_2_O_2_ (1 mM, 10 min). (**d**) PolyADP-ribosylation retained the binding of r-PARP1 to r-p-Erk2 in the presence of ssDNA. PolyADP-ribosylation of r-PARP1 (200 nM) dose-dependently up-regulated its co-immunoprecipitation with r-p-Erk2 (200 nM) in the presence of ssDNA (400 nM) and βNAD. Representative results of 3 experiments are displayed. (**e**) ***Left***: A reduced expression of *c-fos, zif268* and *arc* measured by RT-PCR in cultured rat cerebral neurons stimulated by NGF (60 ng/ml) under hypoxia (100% Argon, 60 min; black), relative to their expression at normal atmosphere (grey). Pre-treatment with gallotannin (100 μM, 60 min) retained their expression under hypoxia. ***Right***: Single strand DNA-breaks in cerebral neurons exposed to hypoxia (100% Argon, 60 min). Each value represents the mean relative expression rate measured in 4 reactions performed in each of 3 experiments.

The expression of *cfos* and *zif268* was measured by RT-PCR in cerebral neurons of PARP1- KO mice that were stimulated (100 Hz, 1 sec, 3 repeats, 10 sec pause) 72 hours after transfection with GFP-fusion vectors with constructs encoding full length PARP1 or PARP1 lacking its DNA binding domain (aa1-221; Methods). Expression of *c-fos* and *zif268* was measured in the re-plated GFP-labeled transfected cerebral neurons (Methods). Cerebral neurons of PARP1-KO mice hardly expressed *c-fos* and *zif268* (Fig. 7a). However, these genes were expressed in stimulated cerebral neurons of PARP1-KO mice transfected with either full length PARP1 or PARP1 lacking its DNA binding domain, evidence that Erk binding domains in PARP1 (but not its DNA binding domain) were necessary for stimulation induced *cfos* and *zif268* expression (Fig. 7a).

**Figure 8:**
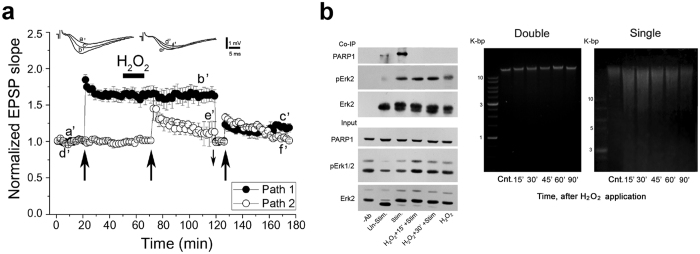
DNA single-strand breaks prevented the generation of LTP. (**a**) Field excitatory postsynaptic potentials (fEPSPs) were recorded from hippocampal slices (5 slices; prepared from 4 male mice). Hippocampal slices were stimulated by the two independent pathway stimulation and recording (Methods). A sample illustration of individual records sampled at the indicated time intervals is presented (**Top**). A train of high-frequency stimulation (100 Hz, 1 sec, denoted with big arrows) was delivered to each pathway. The first stimulation delivered to one of the pathways produced a response of long-term potentiation (LTP). Same stimulation delivered to the second pathway, 15 minutes after application of H_2_O_2_ (1 mM) and 5 minutes washout, failed to produce LTP. Similarly, same stimulation delivered to each pathway 80 mins after H_2_O_2_ application and adjustment of the stimulation intensity to baseline level (downward small arrow) failed to induce LTP of both pathways. (**b**) ***Left:*** DNA single-strand breaks prevented PARP1-Erk2 co-immunoprecipitation in the chromatin of cells prepared from depolarized hippocampal slices (depolarization induced by 1-min wash with ACSF containing 50 mM K^+^). Slices prepared from 10 hippocampi were depolarized, before or after treatment with H_2_O_2_ (1 mM, 15 min, and 5 min washout) inducing single-strand DNA breaks (***Right***).

Some of the transfected PARP1-KO cerebral neurons were treated before stimulation with H_2_O_2_ (1 mM, 10 min) causing single strand DNA breaks (Figs 7c and 8b). As a consequence, the expression of *cfos* and *zif268* was very low in response to stimulation in PARP1-KO neurons transfected with full length PARP1, but was not impaired in PARP1-KO neurons transfected with PARP1 lacking its DNA binding domain (Fig. 7a). Thus, PARP1 binding to nicked DNA was required for preventing *cfos* and *zif268* expression in the presence of single-strand DNA breaks.

In compliance, a brief pre-incubation of cultured rat cerebral neurons with H_2_O_2_ (1 mM; 10 min), or their exposure (60 min) to hypoxia causing DNA single-strand breaks (Fig. 7c,e) down-regulated *cfos* and *zif268* expression and the synthesis of proteins/ transcription factors c-Fos, Zif268 and Arc following high-frequency stimulation (Fig. 7b,e).

Protein synthesis was monitored in stimulated cerebral neurons without or following treatment with H_2_O_2_. These neurons were stimulated by electrical stimulation (3 repeats of 100 Hz, 1 sec duration, each followed by 10 sec pause), without or following treatment with the nerve growth factor NGF ( 60 ng/ml, 5 min) (Fig. 7b). The effect of NGF, also inducing *cfos* and *zif268* expression^17^, was examined because electrical stimulation was technically impossible under hypoxia (Fig. 7b,e).

Treatment with H_2_O_2_ causing single strand breaks, extensively attenuated the stimulation-induced synthesis of proteins c-Fos, Zif268 and Arc, unless cerebral neurons were pre-treated with the PARG (polyADP-ribose glycohydrolase) inhibitor gallotannin (100 μM, 60 min)^48^ (Fig. 7b).

PARG cleaves the negatively charged ADP-ribose polymers of PARP1, enabling its recurrent binding to the negatively charged DNA^2^,^48^. Thus, PARG inhibition interferes with PARP1 binding to nicked DNA^2^,^48^.

Assuming that polyADP-ribosylation does not prevent the binding of PARP1 to phosphorylated Erk2 (Figs 3,4 and 5a), application of PARG inhibitors might preserve the binding of PARP1 to phosphorylated Erk2 by preventing PARP1 binding to single-strand DNA breaks (Fig. 7b). This assumption was examined in a cell-free system by measuring the dose-dependent effect of recombinant PARP1 polyADP-ribosylation on its binding to recombinant phosphorylated Erk2 in the presence of nicked DNA and βNAD (Fig. 7d).

The results indicated that binding of recombinant PARP1 to recombinant phosphorylated Erk2 in the presence of nicked DNA (ssDNA) was dependent on the intensity of PARP1 polyADP-ribosylation (Fig. 7d). The more intensely was PARP1 polyADP-ribosylated, the better it co-immunoprecipitated with phosphorylated Erk2 in the presence of ssDNA (Fig. 7d). This result complied with the preserved expression of *cfos, zif268* and *arc* in the presence of nicked DNA in stimulated cerebral neurons treated with gallotannin (Fig. 7b,e).

### Single-strand DNA breaks prevented LTP generation

Treatment causing DNA single strand breaks exclusively prevented LTP generation in stimulated hippocampal CA3-CA1 connections (Methods; Fig. 8). Notably, the baseline response was not affected, nor already generated LTP (Fig. 8a).

LTP generation in response to a brief high frequency stimulation of the Schaffer collaterals (100 Hz, 1 sec) was exclusively prevented by treatment inducing single-strand breaks (Fig. 8a).

The binding of phosphorylated Erk2 to PARP1 in cell nuclei prepared from the hippocampal slices was also prevented under these conditions (Fig. 8b). PARP1-Erk2 binding was examined in hippocampal slices briefly stimulated by high K^+^ induced depolarization^49^,^50^. This stimulation was used to enhance biochemical processes induced by the tetanic stimulation of the Schaffer collaterals^21^,^22^,^23^. PARP1-Erk2 co-immunoprecipitation was measured in cell nuclei prepared from hippocampal slices briefly exposed to high K^+^ (1 min wash with 50 mM K^+^ ACSF^50^), before and following treatment with H_2_O_2_ (1 mM; 15 min) (Fig. 8b). PARP1 co-immunoprecipitated with phosphorylated Erk2 only in the chromatin of stimulated hippocampal slices that were not treated with H_2_O_2_ (Fig. 8b). This result is consistant with depolarization-induced PARP1-Erk2 binding prevented by treatment causing DNA single strand breaks, which also prevented LTP generation in response to high frequency stimulation (Fig. 8a).

## Discussion

*Ex vivo* and *in vivo* experiments implicated Erk2-induced expression of specific immediate early genes in synaptic plasticity and long-term memory^25^,^26^,^27^,^28^,^36^,^51^. Our results suggest that Erk2-induced PARP1 activation mediates this activity of Erk. These results comply with the dependence of long-term memory acquisition during training on PARP1 activation^13^,^14^,^15^,^16^,^17^,^18^.

PARP inhibition did not affect excitatory post-synaptic NMDA currents (Fig. S1), inducing LTP in the hippocampal CA3-CA1 connections^21^,^23^. However, PARP1 was implicated in nuclear processes immediately following high-frequency stimulation inducing synaptic potentiation. These processes were examined in a model system of electrically stimulated cultured cerebral neurons^29^,^30^ (Fig. S3). High frequency stimulation induced binding of phosphrylated Erk2 to PARP1 in the chromatin of cerebral neurons (Figs 3 and 4), concomitantly with Erk-induced PARP1 activation^9^ (Figs 3b and 6, S6), polyADP-ribosylated linker histone H1 (Fig. 4b), and facilitated access of PARP1-bound phosphorylated Erk2 and acetylated histone H4 to promoters of immediate early genes *cfos* and *zif268*^25^,^26^,^27^,^28^ (Fig. 4a). PARP1-dependent access of phosphorylated Erk2 and acetylated H4 to the promoters of *c-fos* and *zif268* and to their transcription factors Elk1 and CREB were identified by ChIP assay (Fig. 4). These results complied with PARP1-dependent IEG expression^34^,^35^,^36^,^37^,^38^ (Figs 2 and 7a).

In accordance, *cfos, zif268* and *arc* expression was suppressed after PARP1 inhibition or its genetic deletion in cerebral neurons of PARP1-KO mice (Figs 2,4 and 7a). Furthermore, LTP was not generated after PARP1 or MEK inhibition (Fig. 1 and S2), or after PARP1 genetic deletion in the hippocampal CA3-CA1 connections of PARP1-KO mice (Fig. 1c), which indeed do not acquire long-term memory during training^14^.

Thus, PARP1-Erk binding promoting IEG expression (Figs 2, 3, 4 and 7a,d), promoted the forthcoming protein synthesis implicated in synaptic plasticity or long-lasting synaptic potentiation^21^,^23^,^51^. Recent findings indicating protein synthesis during the early phase of high-frequency induced LTP^52^ may support gene expression during early LTP. However, these results do not exclude extra-nuclear processes implicated in early LTP^23^.

Notably, LTP failed to develop in response to high frequency stimulation after treatment causing single strand DNA breaks preventing PARP1-Erk2 binding (Fig. 8), and abrogating the expression of *c-fos* and *zif268* due to a predominant binding of PARP1 to single strand DNA breaks occluding its potential Erk binding sites (Figs 5, 6a and 7a,d).

Furthermore, LTP generated in response to high frequency stimulation 5–10 min before application of MEK or PARP1 inhibitors, was maintained intact (Figs 1 and S2). Moreover, generated LTP remained intact despite producing DNA single-strand breaks (Fig. 8). These results suggest that the brief effects of PARP1-Erk2 binding and their synergistic activity in the chromatin were required for the forthcoming LTP generation (Fig. S7).

The interference of DNA single strand breaks with IEG expression (Fig. 7) may attribute the previously observed aging-induced attenuation in gene expression^53^ to the accumulation of DNA single strand breaks in aged irreplaceable neurons^19^,^20^,^53^,^54^.

The DNA of mammalian cerebral neurons is constantly exposed to damaging processes, mostly by reactive oxygen species (ROS), which are normally produced in their mitochondria due to high-energy demands^55^. ROS cause single strand DNA breaks by oxidative reactions with the nucleic acids^55^. Thus, age-induced decline of antioxidant defensive mechanisms, the inability to replace aged neurons and the constant exposure of their DNA to oxidative stress during their life span, cause accumulation of single strand breaks in the DNA of cerebral neurons in senescence, despite the existing DNA repair mechanisms^19^,^20^.

Single strand DNA breaks interfering with IEG expression under hypoxia (Fig. 7e) could be implicated in the negative effects of hypoxia on synaptic plasticity in the hippocampus^20^.

Furthermore, failure to generate LTP due to accumulating DNA single-strand breaks in aged cerebral neurons (Fig. 8) could be implicated in the deterioration of memory acquisition and learning abilities, frequently experienced in senescence^19^,^20^,^53^. Thus, deterioration in learning abilities might not necessarily reflect death of cerebral neurons. It could result from the accumulation of amendable single-strand DNA breaks in aged irreplaceable cerebral neurons interfering with LTP generation (Figs 6, 7, 8). In this case, memory acquisition could be improved by attenuating the binding of PARP1 to nicked DNA (Figs 7 and 8). In support, recent evidence indicated an improved long-term memory acquisition of aged mice treated with the PARG inhibitor gallotannin^48^,^56^.

Notably, PARP1 inhibitors impaired long-term memory acquisition of trained animals only when administered at least 30 min before training^13^,^15^. Their application after training did not affect the already acquired memory of the trained animals^13^. Similarly, PARP inhibitors prevented the induction of LTP only when applied before stimulation (Fig. 1e,f). These findings may suggest a possible use of PARP1 inhibitors for erasing a specific memory without affecting past memories or learning abilities.

In summary, the presented findings disclose a molecular mechanism in the chromatin of cerebral neurons, which is necessary for LTP generation, and can be manipulated by pharmacological interventions.

## Methods

### Antibodies and recombinant proteins used in the presented experiments

PARP1 and its recombinant domains were immunolabeled by the monoclonal antibody (Serotec, Cat # MCA1522; Oxford, UK) and the polyclonal antibody (Alexis, Cat # ALX210-302). Erk2 was immunolabeled by antibody directed against the c-terminal of Erk2 (#sc-154; Santa Cruz Biotechnology, CA, USA), phosphorylated Erk1/2 (Sigma), Elk1 and phosphorylated Elk1 (Cell Signaling Technologies, MA, USA). Antibodies directed against acetylated histones H3 and H4 were from Upstate Biotechnology (Millipore) CA, USA. Antibodies directed against transcription factors c-Fos (Cell Signaling Technologies), Egr1 (Zif268; Cell Signaling Technologies), phosphorylated CREB (phosphorylation of serine-133; Cell Signaling Technologies) and Arc (Novous Biologicals, Cambridge, UK). For cytochemistry, first antibodies were labeled by fluorescent secondary antibody: CyTM2 (green) or fluorescent CyTM3 (red) conjugated affinity pure goat-anti-rabbit or goat anti-mouse secondary antibodies (Jackson ImmunoResearch). Recombinant proteins: Elk1 (Elk1 residues 307–428 coupled to GST; Cell Signaling Technologies), recombinant human PARP1 was commercial (Alexis, Enzo Life sciences, NY,USA) or prepared by Dr John Pascal, Thomas Jefferson University, Philadelphia, USA, recombinant PARP1 domains were prepared in the lab of Dr Francoise Dantzer (Strasbourg, France) recombinant PARP1 (1-494aa) was prepared in the lab of Dr. John Pascal, as well as constructs in a GFP fusion vector of full-length PARP-1 and PARP-1 residues 201- 1014aa for expression in cultured neurons of PARP1 KO mice. Recombinant phosphorylated Erk2 was prepared in the lab of Prof Seger, Weizmann Institute of Science, Rehovot.

**Primary cell cultures** were prepared from brain cortex and hippocampus (cerebral neurons) of 18 to 19 day rat or mice embryos, as described before^57^. Experiments were conducted according to rules and regulations of Institutional Animal Care and Use Committee.

#### Nuclear protein extracts

Cell nuclei were isolated from cultured cerebral neurons as described before^57^. Nuclear proteins were extracted after incubation (30 min on ice) in a high salt concentration buffer, containing 0.42 M NaCl, 1.5 mM MgCl_2_, 0.2 mM EDTA, 25% glycerol, 20 mM Tris-HCl pH 8.0, protease and phosphatase inhibitors. Supernatants obtained after centrifugation (15,000 rpm 4 °C, 15 min) contained extracted nuclear proteins.

#### Electrophysiology in hippocampal slices

The methods of recording from hippocampal slices were described before^23^,^58^. Briefly, male 129/Sv mice (2–2.5 month-old) were rapidly decapitated and their brains were removed and placed in ice cold ACSF containing (mM) 124 NaCl, 2 KCl, 26 NaHCO_3_, 1.24 KH_2_PO_4_, 2.5 CaCl_2_, 2 MgSO_4_ and 10 glucose, at pH7.4. The hippocampi were cut into 350–400 μm transverse slices using a McIlwain tissue chopper. Slices were incubated for 1.5 h in carbogenated (5% CO_2_ and 95% O_2_) ACSF at room temperature in a holding chamber. Recording was made from slices that are slightly submerged in a standard chamber at 33.8–34.0 °C with a flow rate of 2.5 ml ACSF/min. Field excitatory postsynaptic potentials (fEPSPs) were recorded in stratum radiatum of the CA1 region of hippocampal slices through a glass pipette containing 0.75 M NaCl (4 MΩ). Synaptic responses were evoked by stimulation of the Schaffer collaterals using two sets of bipolar electrodes placed on both sides and equidistant from the recording pipette, such that two independent stimulation channels were used for each slice^23^,^58^ (Fig. 1a). LTP was induced by high-frequency stimulation (100 Hz, 1 sec). Before applying the stimulation, evoked fEPSPs (50% of maximum amplitude) were recorded for a stable baseline period of at least 10 min. Stimulation of one pathway did not cause any noticeable change in response to stimulation of the second pathway, verifying their independence^58^. Data acquisition and off-line analysis were performed using pCLAMP 9.2 (Axon Instruments, Inc). All numerical data are expressed as mean ± SEM, and fEPSP slope changes after stimulation and drug application were calculated with respect to baseline. PARP1(−/+) 129/Sv mice were donated by Dr Dantzer (Strasbourg) and bred for PARP1 (−/−) mice in Cohen-Armon’s lab under the rules and regulations of the Institutional Animal Care and Use Committee.

The effect of H_2_O_2_ on LTP was examined as follows: After 20 minutes of baseline recording, first tetanic stimulation (100 Hz, 1 sec) was applied to pathway 1, which resulted in LTP of a magnitude of 1.64 ± 0.004. H_2_O_2_ (at final concentration in ACSF 1 mM, Sigma Aldrich) was added for 15 minutes after potentiated pathway has stabilized. Its application did not affect either magnitude of already established LTP or baseline responses of pathway 1. Following 5 minutes of washout of H_2_O_2_, a tetanus was delivered to pathway 2 to investigate H_2_O_2_ impact on LTP induction. Post-tetanic long-term potentiation failed to develop (LTP 1.12 ± 0.01, p < 0.001 was measured). After 1 hour of recording and adjustment of stimulation intensity of both pathways to baseline level, a second tetanic stimulation was applied to each pathway. LTP did not developed in both pathways (LTP 1.19 ± 0.01 ;p < 0.001 was measured in pathway 1 and 1.05 ± 0.01 p < 0.05 in pathway 2, correspondingly).

**Electrical stimulation (‘bath stimulation’) of cerebral neurons in primary culture** was applied by a pulse generator controlled by pCLAMP 6.0 (Axon Instruments, Inc.) and a digital to analogue conversion (D/A 1200 Digidata), as described before^7^. Usually, train of pulses (0.5 msec) of 1 sec duration, 1–100 Hz frequency, was repeated 3 times, each followed by 10 sec pause intervals. Pulse amplitude was the minimal voltage required for a break of action potential in randomly chosen neurons in the culture, as described before^7^. About 30 Volt was applied to the bath solution (5 ml growth medium containing MEM eagle enriched by 2 mM Glutamax, 0.6% glucose, 5% Horse Serum, 20 μg/ml gentamycin). This stimulation caused pre-synaptic vesicles recycling (Fig. S3).

#### Culturing neurons on glia cells

Mouse postnatal cultures were platted on glia cells prepared from rat E19 embryos, as detailed before^59^. Glia cells proliferated for 10 days before plating the mouse culture. This procedure was used for re-plating of transfected neurons and for plating cerebral neurons of PARP1 KO mice.

#### PARP1 activation in cerebral neurons

Two-dimensional (2-D) gel electrophoresis was used to identify stimulation-induced activation of the positively charged DNA-binding protein PARP1 by the shift in its isoelectric point (pI) towards lower pH, due to polyADP-ribosylation adding negatively charged phosphates to PARP1. This method was used to estimate PARP1 activation *in situ*^13^,^15^,^57^. In support, PARP1 activation was measured by the shift in the pI of [^32^P]polyADP-ribosylated PARP1 in isolated nuclei of stimulated cerebral neurons incubated with [^32^P]NAD (1 μCi/sample; 1000 mCi/mmol; Amersham, UK) (Fig. S4).

#### For RT-PCR profiling

we used RNeasy Plus mini kit (Qiagene, CA, USA) for RNA preparation, and we used RevertAid First Strand cDNA Synthesis Kit #K1622 (Thermo scientific) for cDNA preparation. Primers that initiated amplification of the indicated cDNA segments in the rat genes *cfos, Zif268* and *arc* (forward and reverse) were: for *c-fos*, 5′GTTCCTGGCAATAGTGTGTTC3′ and 5′GCTGAAGAGCTACAGTACGTG3′, for *arc*, 5′TGGAGTCTTCAGACCAGGTG3′ and 5′GCTGGCTTGTCTTCACCTTC3′, for *zif268*, 5′CAGGAGTGATGAACGCAAGA3′ and 5′AGCCCGGAGAGGAGTAAGTG3′, for *c-jun1*, 5′TGAGAACTTGACTGGTTGCG3′ and 5′CAGGTGGCACAGCTTAAACA3′. For the control gene GAPDH; 5′CTGGAAAGCTGTGGCGTGATGG3′ and 5′TCCTCAGTGTAGCCCAG GATGC3′ and for the control gene *β-actin,* 5′AGAGCTATGAGCTGCCTGAC3′ and 5′AATTGAATGTAGTTTCATGGATG3′.

Primers that initiated amplification of the indicated cDNA segments in the genes *c-fos, zif268* and *arc* (forward and reverse) in mice were: *c-fos* forward 5′TCCGGGCTGCACTACTTA3′ and reverse 5′TGTTTCACGAACAGGTAAGGT3′, respectively. For *zif268*, 5′GTGTGGTGGCC TCCCCGGCT3′ and 5′CACTGACGGCGACGGGAAGCC3′, respectively. For *arc,* 5′GCCACAAATGCAGCTGAAGCAG3′ and 5′GTGGTGTGGTGATGCCCTTTCC3′, respectively. For the control gene *β-actin* forward 5′GGGCTGTATTCCCCTCCAT3′ and reverse 5′GCGTGAGGGAGAGCATAGC3′, respectively.

#### Chromatin immunoprecipitation (ChIP) assay

We used the ChIP assay protocol^5^ to identify binding of phosphorylated Erk2 and Acetylated H4 (AcH4) to promoters of *c-fos*, and *zif268*. DNA-bound proteins were crosslinked to the DNA of stimulated mice cerebral neurons (by formaldehyde 1%) at different intervals after stimulation. The crosslinked chromatin was cleaved into segments of approximately 1000-bp by sonication on ice (Probe Sonicator; Heat Systems Inc., Farmingdale, USA). Promoters and transcription factors were co-immunoprecipitated by antibody directed against AcH4 (#06-866 anti-acetyl-H4 antibody directed against epitope aa2-19 in H4 acetylated on lysines 5,8,12,16; Upstate Biotechnology (Millipore) CA, USA), or by antibody directed against the c-terminal of Erk2 (#sc-154; Santa Cruz Biotechnology, CA, USA). Both DNA and proteins were recovered from the crosslinked chromatin segments after co-immunoprecipitation as described before^5^. For DNA isolation, formaldehyde cross-linking was reversed by heating (65 °C, 2 h). After protein digestion, DNA was purified on Zymo-Spin^TM^ columns (ChIP DNA Clean & Concentrator kit, Zymo research corp.). DNA segments in the promoters were amplified by RT-PCR by using the following primers: for the promoter of *c-fos*, primers 5′GTGCTGCCGTCCTTTAAAAC-3′ and 5′-GAGAGAGGGGCTGAGAAGCT-3′ (amplified segment 60–204) and primers 5′CTGCACTGATTTGGGATGGG-3′ and 5′-TAGGAGAAGCAAGTACGCAGC3′ (amplified segment 98–150). For the promoter of *Zif268* 5′-TGGGGCTCCCGAAATACAAC-3′ and 5′-AAGAGGGGGACTTGGCTTTG-3′ (amplified segment 382–395), and primers 5′-AGGACGGAGGGAATAGCCTT-3′ and 5′-ACTGGTTC TTGGGACACTGC-3′ (amplified segment 659–787). Proteins were recovered from the croslinked chromatin after 15 boiling in sample buffer.

#### Expression of PARP1 in PARP1-KO cerebral neurons

Cerebral neurons of PARP1-KO mice were transfected 24 hours after plating with two plasmids (in mammalian expression vector (pEGFP-N1) encoding GFP-fusion full-length PARP1 or GFP-fusion PARP1 lacking residues aa1-201 (lacking the DNA binding zinc fingers domain), which were prepared in Dr John Pascal Lab (Jefferson University, Philadelphia). The expression of *c-fos* and *zif268* was measured by RT-PCR in transfected GFP-labeled KO cerebral neurons sorted by FACS, 60–72 hours after transfection. The transfected neurons were re-plated on cultured rat glia cells and stimulated by bath stimulation, without or after treatment with H_2_O_2_ (1 mM, 10 min).

#### DNA isolation and detection of DNA breaks

DNA was isolated from the nuclei of cultured neurons using the PureLink genomic DNA kit (Invitrogen, Cat # K1820-01). Single strand DNA breaks were identified on alkali agarose gels containing 1% agarose, 50 mM NaCl, 1 mM EDTA, soaked for 60 min with 30 mM NaOH and 1 mM EDTA, as described before^7^. Double strand DNA breaks were detected in 1% agarose gel at pH 7.4. The migration of DNA in 1% agarose gel was detected by staining under UV illumination. In cell-free experiments we used commercial ssDNA (salmon sperm DNA) carrying numerous single strand breaks (Sigma).

#### PARP1 silencing by siRNA

This method was described before^5^,^9^. Two sequences, aa800-807 and aa890-897, in the PARP1 catalytic domain were targeted for PARP1 silencing. PARP1 targeted siRNA was prepared by Darmacon (Lafayette CO, USA). For control we used the non specific siRNA#2 (non-spec. rat siRNA; Darmacon). Cerebral neurons were transfected by XtremeGENE siRNA transfection reagent (Cat no. 04476093001, Roche Diagnostic, GmbH Mannheim, Germany). PARP1 silencing was achieved 72 hours after transfection with 100–200 nM siRNA.

### Bioinformatic analysis of PARP1 binding to phosphorylated Erk2

Identified docking sites of Erk on the F-domain of PARP1^41^,^42^,^43^,^44^: Phosphorylated Erk2 homodimer^47^ was reconstructed from the crystal contact interface in PDB (Protein Data Bank). Phosphorylated Erk2 homodimer was docked on the helical, catalytic and WGR domains of PARP1 (PDB 4DQY). Details are included in Supplementary Methods.

#### Treatment with H_2_O_2_

Cerebral neurons in cell culture (10 days after plating), and hippocampal slices were exposed to H_2_O_2_ (1 mM, 10–15 min), and then thoroughly washed, as described before^7^.

#### Cerebral neurons under hypoxia conditions

Cultured rat cerebral neurons were exposed after over-night starvation (MEM-Eagle growth medium containing 0.5% Horse serum instead of 5% in normal growth medium, 0.6% glucose, 2 mM Glutamax and 20 μg/ml Gentamycin) to hypoxia at 37 °C for 60 min. Hypoxia was imposed after replacing the normal atmosphere in a close chamber with 100% Argon. A similar procedure was described before^60^.

#### Co-immunoprecipitation

was used to identify bound recombinant proteins or nuclear proteins as described before^5^,^9^. Binding to specific antibodies trapped the proteins on Protein A/G Agarose Beads (1 h, 4 °C). The bound proteins were recovered (1–2 min, boiling in sample buffer) separated on polyacrylamide SDS gel and immunodetected on Western blots.

### Dot Blot analysis searching PARP1 domains binding phosphorylated Erk2

Binding of recombinant phosphorylated Erk2 (1 μg) to recombinants of PARP1 and to recombinant domains of PARP1 was examined by dot-blot analysis. In addition, binding of phosphorylated Erk2 to polyADP-ribosylated recombinant human PARP1 and free [^32^P]-labeled poly(ADP-ribose were examined. The blots were blocked in ‘binding buffer’ (50 mM Tris-HCl pH7.5, 120 mM NaCl, 0.1% NP40, 0.5 mM PMSF, 20 mg/ml BSA) for 30 min at room temperature. These blots were incubated for 3 h at room temperature in ‘binding buffer’ containing 5 μg/ml of each of the purified recombinant: human PARP1, recombinant domains of human PARP1, polyADP-ribosylated human PARP1, or 2 μg/ml of free [^32^P]polyADP-ribose polymers. The blots were then washed with TBS-Tween-20 0.1%. Anchored proteins were detected on the nitrocellulose membrane with the appropriate antibodies. Binding to free [^32^P] poly(ADP-ribose) was measured by autoradiography.

All the methods were carried out in accordance with the approved guidelines. All experimental protocols were approved by the Institutional Animal Care and Use Committees of the Sheba Medical Center and the Tel-Aviv University.

## Additional Information

**How to cite this article**: Visochek, L. *et al*. A PARP1-ERK2 synergism is required for the induction of LTP. *Sci. Rep.* **6**, 24950; doi: 10.1038/srep24950 (2016).

## Supplementary Material

Supplementary Information

Supplementary Movie S1

Supplementary Movie S2
