# Supplementary material for: A PARP1-ERK2 synergism is required for the induction of LTP
Source: Sci Rep. 2016 Apr 28;6:24950. doi: 10.1038/srep24950 (PMC4848477; doi:10.1038/srep24950)
Supplement: Supplementary Information [file srep24950-s1.pdf]

Supplemental information

A PARP1-ERK2 synergism is required for the induction of LTP

L. Visochek, G. Grigoryan, A. Kalal, H. Milshtein-Parush, N. Gazit, I. Slutsky, A. Yeheskel, A. Shainberg, A. Castiel, R. Seger, M.F. Langelier, F. Dantzer, J. Pascal, M. Segal, M. Cohen-Armon.

Supplemental Results

To examine possible effect of PARP1 inhibitors on excitatory postsynaptic NMDA current, evoking LTP in hippocampal CA3-CA1 connections[23], NMDA currents recorded from depolarized cells in the CA1 pyramidal cell layer were measured, before and after application of PJ-34 and ABT-888 at concentrations affecting LTP (Fig 1, e and f). The currents were measured in response to low frequency stimulation, because high frequency typically induces rundown of NMDAR-mediated EPSC (Fig S1).

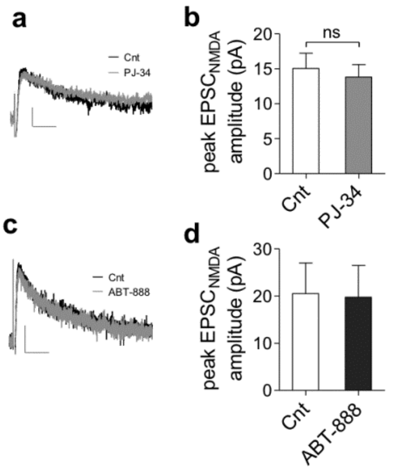

Figure S1. PARP1 inhibitors PJ-34 (20 μM) and ABT-888 (20 μM) did not block excitatory NMDA postsynaptic currents (EPSC$_{NMDA}$) measured by whole cell patch-clamp of CA1 pyramidal neurons in hippocampal slices. (**a, c**) Representative recordings of EPSC$_{NMDA}$s in CA1 pyramidal neurons (holding potential, +40 mV) evoked by low frequency stimulation (0.1 Hz) of Schaffer Collaterals in acute hippocampal slices under control conditions (Cnt) and 10 min following application of PJ-34 (a) or ABT-888 (c). Scale bars: 100ms, 5pA (PJ-34);

100ms, 10pA (ABT-888). (**b, d**) Peak $EPSC_{NMDA}$ amplitude is not altered by PJ-34 (n = 4, p > 0.05, **b**) or ABT-888 (n = 3, p > 0.05,**d**).

MEK inhibition prevented LTP induction without impairing already established LTP

Field excitatory postsynaptic potentials (fEPSPs) were recorded from hippocampal slices (n=4, prepared from 2-month-old C57BL/6 male mice (n=3)). High frequency stimulation (100 Hz, 1 sec) was induced using two sets of bipolar electrodes placed on both sides of the slide and equidistant from the recording pipette, such that two independent stimulation-recording channels were used for each slice (Methods). After 20 minutes of baseline recording, stimulation was applied to one of the pathways, which resulted in a stable LTP of a magnitude of 1.52 ± 0.01, recorded for 120 min. In contrast, same stimulation delivered to the second pathway after 50 min perfusion of the MEK inhibitor PD98059 (50 µM, AdipoGen) failed to generate LTP (average values measured for 60 min after stimulation, 1.15 ± 0.01 above baseline; Fig S2). LTP generated 10 min before application of the MEK inhibitor PD98059 was not impaired.

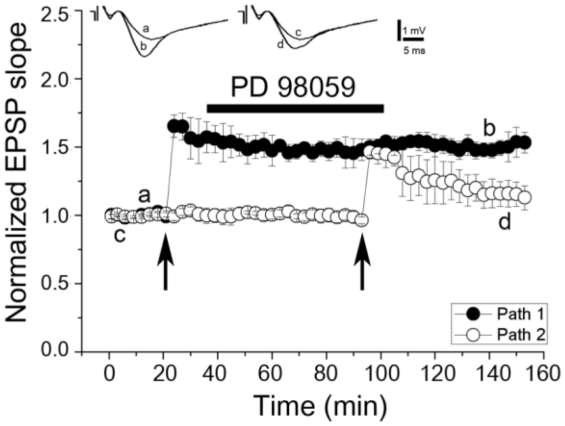

Figure S2. LTP was not generated in hippocampal CA3-CA1 connections after MEK inhibition.

A sample illustration of individual records sampled at the indicated time intervals is presented (**Top**). A train of high-frequency stimulation (100 Hz, 1sec, denoted with arrows) was

delivered to each of 2 independent stimulation-recording pathways. The first stimulation delivered to one of the pathways induced a response of long-term potentiation (LTP). Same stimulation delivered to the second pathway, 50 minutes after application of the MEK inhibitor PD98059 (50 µM), failed to produce LTP, but did not impair the already generated LTP.

Three repeats of high frequency stimulation inducing LTP in hippocampal slices (Fig 1) induced long-term synaptic potentiation, indicated by pre-synaptic vesicle recycling in cultured cerebral neurons (Figure S3).

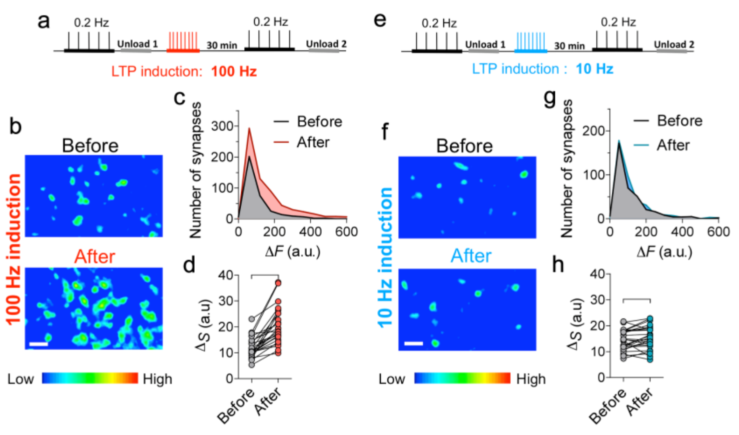

Figure S3. Long-term synaptic potentiation indicated by synaptic vesicle recycling in response to stimulation in cultured cerebral neurons

a. The experimental protocol used to measure synaptic vesicle recycling induced by low-frequency stimulation (0.2 Hz) before and after LTP induction protocol consisting of 3 repeats of 100 Hz during 1sec, interval of 10 sec.

b. Representative images before and 30 min after LTP induction by 100 Hz stimulation. Scale bar: 2 µm.

c. Δ*F* histograms before (grey) and after LTP induction at 100 Hz (red) in a single experiment. The median fluorescence intensities (Δ*F*) increased from 75 to 109 a.u. and the number of synapses increased from 344 to 676.

d. Average increase by 70% in the presynaptic strength (ΔS = Δ*F* x *D*) by 100 Hz stimulation in 6 experiments (P < 0.0001).

e. The experimental protocol used to measure synaptic vesicle recycling induced by low-frequency stimulation (0.2 Hz) before and after LTP induction protocol consisting of 3 repeats of 10 Hz during 1sec, interval of 10 sec.

f. Representative images before and 30 min after LTP induction by 10 Hz stimulation. Scale bar: 2 µm.

g. Δ*F* histograms before (grey) and after LTP induction at 10 Hz (blue) in a single experiment. The median Δ*F* changed from 77 to 81 a.u. and the number of synapses from 361 to 389.

h. On average, the presynaptic strength (ΔS) was not affected by 10 Hz stimulation in 6 experiments ($P > 0.05$).

Supplement to Fig 2.

c-Jun1 was scarcely expressed in cultured rat cerebral neurons stimulated by high frequency stimulation inducing expression of *c-fos, zif268* and *arc* (Fig 2).

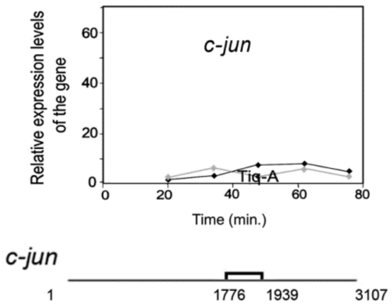

Figure S4 The relative expression rate of *c-jun1* was measured in cultured rat cerebral neurons by RT-PCR at the indicated time intervals following stimulation (3 repeats of 1 sec, 100 Hz, each followed by 10 sec pause). Its expression rate was related to the expression rates of two reference genes (*β-actin* and *GAPDH*) that are not expressed in response to the applied stimulation. The relative expression of *c-jun1* in response to stimulation was measured without (black line) or in the presence of the PARP inhibitor Tiq-A (50 µM) (grey lines). Each value represents the average value of 4 reactions (with calculated variation coefficient) in each of 3 experiments.

Supplement to Fig 3.

Two methods for measuring PARP1 activation were compared. We found that PARP1 activation inducing its polyADP-ribosylation can be measured by the shift in its isoelectric point (pI) towards

acidic pH. The pI of PARP1 in un-stimulated neurons (about pH 9.5 ; Fig 3) was shifted towards lower pH, due to negatively charged ADP-ribosyl moieties forming polymers on activated PARP1 in the nuclei of NGF treated neurons.

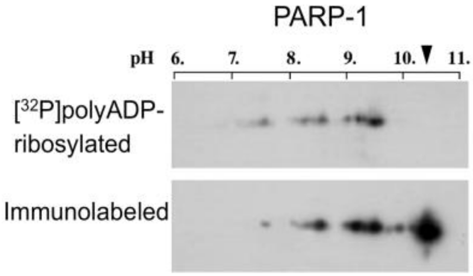

Figure S5. The isoelectric point (pI) of [^{32}P]polyADP-ribosylated PARP1 in isolated nuclei of NGF treated neurons (60 ng/ml NGF, 5 min) was shifted towards acidic pH.

PARP1 activation was measured in parallel by its [^{32}P]polyADP-ribosylation in nuclei exposed to [^{32}P]NAD (1μ Ci/sample; 1000 mCi/mmol) and by its shifted pI**.** The isoelectric point of [^{32}P]polyADP-ribosylated PARP1 was shifted from pH 9.5 (before stimulation) towards pH 7.5 in neurons treated with NGF. [^{32}P]polyADP-ribosylated PARP1 was auto-radiographed and immunolabeled (Serotec, Cat # MCA1522). Representative results obtained in 3 different experiments are displayed.

Supplement to Fig 6.

Figure S6. Calculated intra-molecular movements (directions of motion) in PARP1 bound to either phosphorylated Erk2 or DNA.

The anisotropic network model (ANM) was used to predict the directions of motions in PARP1 bound to phosphorylated Erk2 and in PARP1 bound to DNA (Supplementary Methods). The helical (HD) and the catalytic (CAT) domains of PARP1 moving to opposite directions (yellow arrows) in PARP1 bound to phosphorylated Erk2 expose its NAD binding site. This may underlie polyADP-ribosylation of Erk-bound PARP1[9] (Figs 3, 6C, 7d). The NAD binding site is not similarly exposed by the same calculated movement in DNA-bound PARP1.

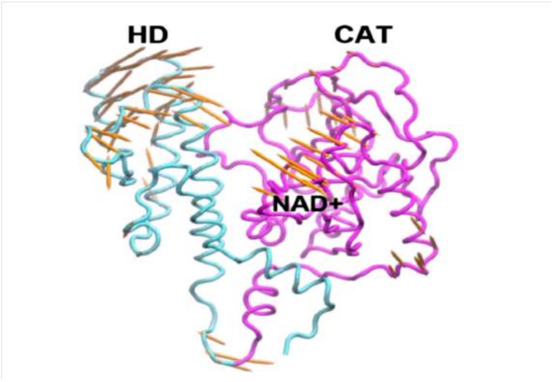

Movie1:PARP1 bound to homodimer of phosphorylated Erk2

78357_0_video_927004_nbh69c.mpg

Movie2: PARP1 bound to DNA

78357_0_video_927005_nbh69c.mpg

Figure S7.

A schematic presentation of the estimated timing of the identified processes, induced by high frequency stimulation of cerebral neurons (Figures 2-7)

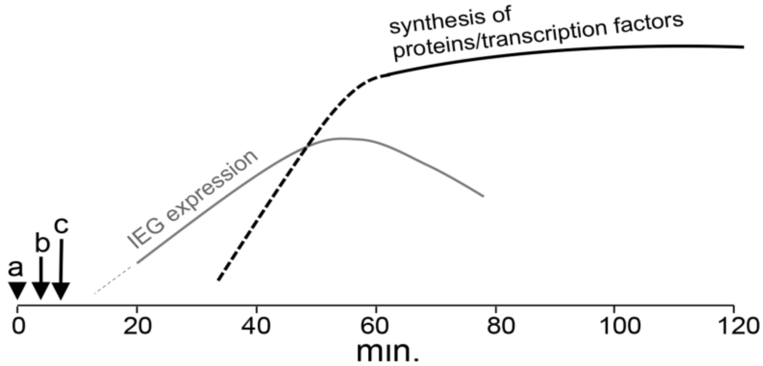

a. High frequency electrical stimulation
b. PARP1 binding to phosphorylated Erk2 translocated to the nucleus
c. PARP1 mediated phosphorylation of transcription factors (Elk1,CREB)

IEG: ***cfos, zif268, arc***

Supplemental Methods

Measurement of NMDA currents in acute hippocampal slices

Wild-type mice (C57BL/6J and balb/c) were used. Coronal slices (350 μm) of hippocampus were prepared as described. Slices were transferred to a submerged recovery chamber at room temperature (21–23°C) containing oxygenated (95% O_2 and 5% CO_2) artificial cerebrospinal fluid (ACSF) for 1 h before the experiment. The ACSF contained, in mM, NaCl, 125; KCl, 2.5; $CaCl_2$, 1.2; $MgCl_2$, 1.2; $NaHCO_3$, 25; NaH_2PO_4, 1.25; glucose, 25. After 1-4 h of recovery, slices were transferred to the recording chamber on the stage of Olympus BX51WI microscope. Whole-cell patch pipettes (2–4 MΩ) were used to record $EPSC_{NMDA}$s under a +40mV holding potential. The intracellular solution was following (in mM): Cesium Methanesulfonate, 120; HEPES, 10; NaCl, 10; $CaCl_2$, 0.5; EGTA, 10; Mg-ATP, 2; and NaGTP, 0.3; pH adjusted to 7.25 with CsOH. Serial resistance was not compensated. DNQX (20mM, Tocris) and bicuculline (30 μM, Tocris) were added to the ACSF solution to block non-NMDA currents. Stimuli in the Schaffer collateral–commissural pathway in the stratum radiatum evoked EPSCs responses that were recorded from the CA1 pyramidal cell layer.

Measurement of synaptic long-term potentiation based on FM dye staining[1]

Synaptic vesicle release at single synapses in primary neuronal cultures was determined using the activity–dependent FM1-43 dye as described before[1]. Briefly, action potentials (APs) in neurons were initiated by field stimulation during dye loading, and the terminals, after undergoing vesicle exocytosis coupled to endocytosis, were stained by 10 μM FM1-43. During FM loading and unloading the extracellular solution contained (in mM): NaCl, 145; KCl, 3; glucose, 15; HEPES, 10; $MgCl_2$, 1.2; $CaCl_2$, 1.2; pH adjusted to 7.4 with NaOH. Kynurenic acid (0.5 mM) was added to prevent recurrent activity through blockage of excitatory postsynaptic responses during loading and unloading. After dye loading, external dye was washed away in Ca^{2+}-free solution containing ADVASEP-7 (0.1 mM; Sigma). To confirm that the fluorescent spots corresponded to release sites, we evoked at 5 Hz for 4 min during the unloading step to obtain release of dye–filled vesicles. The total amount of

releasable fluorescence at each bouton (ΔF) was calculated from the difference between fluorescence after loading and after unloading ($\Delta F = F_{loading} - F_{unloading}$). The total presynaptic strength has been calculated as $S = \Delta F \times D$, whereas D is the density of FM-(+) puncta per image area. To determine the sign and magnitude of short-term plasticity, we calculated the Sburst / Ssingle ratio over the same image area, whereas Ssnigle was measured for the loading of 30 APs @ 1 Hz and Sburst for 30 APs @ 6 bursts (each burst contained 5 APs, inter-spike interval = 10 ms, inter-burst-interval = 5 sec).

Bioinformatic analysis of PARP1 binding to phosphorylated Erk2 or to DNA

Phosphorylated Erk2 homodimer was docked on the helical, catalytic and WGR domains of PARP1 (PDB 4DQY) using PyDock[2] and Patchdock[3], for the CRS/CD proteins binding region of Erk2 and two of the possible Erk2 binding motifs on PARP1 (aa747-752 and aa633-637). Domains ZN1, ZN3, WGR, HD and CAT of PARP1 were modeled according to PDB 4DQY. Domain ZN2 was modeled according to PDB 3ODC. Domain BRCT was modeled according to PDB 2COK. Docking predictions were selected based on their electrostatic match between Erk2 and PARP1 interfaces. The electrostatic potential regions in PARP1 and Erk2 were calculated using APBS3[4], as implemented in PMV[4,5] and visualized in PyMol[6,7] (The PyMOl Molecular Graphics System, Version 1.3 Schrödinger, LLC). Two methods were used for *in-silico* molecular docking of PARP1 (aa 531-1014) to ERK2 homodimer: PatchDock[2], followed by FireDock[3] for docking refinement, and pydock[4]. The electrostatic potentials of PARP1 (aa531-1014) and ERK2 monomer were calculated using APBS and projected on their molecular surface using UCSF Chimera[6]. Positively charged patches that are predicted to bind Erk2 on PARP1 (residues aa633-637 and aa747-752) were selected for *in-silico* molecular docking (by a computational method that predicts the preferred orientation of two molecules forming a stable complex).

Calculated intra-molecular dynamics (directions of motion) in PARP1 bound to phosphorylated Erk2. The conformational changes of PARP1 and Erk2 were predicted using the anisotropic network model, a normal mode analysis tool available online:

http://ignmtest.ccbb.pitt.edu/cgi-bin/anm/anm1.cgi. The anisotropic network model (ANM) was used to predict the directions of motions in PARP1 bound to phosphorylated Erk2 and in PARP1 bound to DNA. The motion prediction was presented using NMWiz[7], a normal mode analysis plug-in for molecular graphic viewer[8,9] .

Supplementary References

1. Slutsky I., Sadeghpour s., Li B., Liu G. Enhancement of synaptic plasticity through chronically reduced Ca^{2+} flux during uncorrelated activity. *Neuron* **44**, 835-849 (2004).
2. Jimenez-Garcia B., Pons C. and Fernandez-Recio J. *pyDockWEB: a web server for rigid-body protein-protein docking using electrostatics and desolvation scoring. Bioinformatics* **29,** 1698-1699 (2013).
3. Schneidman-Duhovny D, Inbar Y, Nussinov R, Wolfson HJ. PatchDock and SymmDock: servers for rigid and symmetric docking. *Nucl. Acids. Res.* **33,** W363-367(2005).
4. Mashiach E., Schneidman-Duhovny D., Andrusier N., Nussinov R., Wolfson H.J. FireDoc: a web server for fast interaction refinement in molecular docking. *Nuc. Acid. Res*. **36,** W229-W232 (2008).
5. Baker N.A., Sept D., Joseph S., Holst M.J., McCammon J.A. Electrostatics of nanosystems: application to microtubules and the ribosome. *Proc. Natl. Acad. Sci. USA.* **98,** 10037-10041 (2001).
6. Pettersen E.F., et al. UCSF Chimera--a visualization system for exploratory research and analysis. *J. Comput. Chem.* **25,** 1605-1612 (2004).
7. Bakan A., Meireles L.M., Bahar I. ProDy: Protein dynamics inferred from theory and experiments. *Bioinformatics* **27,** 1575-1577 (2011).
8. Humphrey W., Dalke A., Schulten K. VMD: Visual molecular dynamics. *J. Mol. Graph.* **14,** 33-38 (1996).
9. Sanner, M.F. Python: A Programming Language for Software Integration and Development. *J. Mol. Graphics Mod.* **17,** 57-61 (1999).
